# Seroprevalence of *Borrelia burgdorferi* Antibodies in Patients with Ulcerative Colitis and Its Association with Disease Activity

**DOI:** 10.3390/pathogens15040408

**Published:** 2026-04-09

**Authors:** Gokhan Aydin, Taner Akyol

**Affiliations:** 1 Division of Gastroenterology, Department of Internal Medicine, Faculty of Medicine, Giresun University, 28200 Giresun, Turkey; 2 Division of Gastroenterology, Department of Internal Medicine, Faculty of Medicine, Samsun University, 28000 Samsun, Turkey; taner.akyol@samsun.edu.tr

**Keywords:** ulcerative colitis, *Borrelia burgdorferi*, seroprevalence, disease activity

## Abstract

**Background and Aim:** Ulcerative colitis (UC) is a chronic inflammatory bowel disease characterized by immune dysregulation. Environmental factors, including infectious agents, have been proposed to influence disease activity in inflammatory bowel disease. Although *Borrelia burgdorferi* has been shown to exert complex immunomodulatory effects on host immune responses, its seroprevalence and potential association with disease activity in patients with ulcerative colitis have not been systematically investigated. This study aimed to evaluate the seroprevalence of *Borrelia burgdorferi* IgG antibodies in patients with ulcerative colitis and to assess the relationship between seropositivity and laboratory markers of disease activity. **Methods:** In this retrospective observational study, 100 patients with ulcerative colitis (59 males, 41 females; mean age 48.5 ± 17 years) who underwent *Borrelia burgdorferi* IgG serological testing due to musculoskeletal or neurological symptoms suggestive of possible Lyme disease between October 2020 and October 2024 were included. Demographic characteristics, hematological and biochemical parameters, and inflammatory markers were compared between seropositive and seronegative groups. Due to the retrospective design, validated clinical disease activity indices were not consistently available; therefore, disease activity was indirectly assessed using laboratory inflammatory markers. **Results:** Among patients with ulcerative colitis, 22% were seropositive for *Borrelia burgdorferi* IgG. Seropositive patients had significantly lower uric acid, alkaline phosphatase, and C-reactive protein levels compared to seronegative patients (*p* = 0.001, *p* = 0.023, and *p* = 0.020, respectively). Free T4 levels were significantly higher in the seropositive group (*p* = 0.049). In terms of erythrocyte indices, mean corpuscular volume and mean corpuscular hemoglobin were significantly higher, while RDW-CV values were significantly lower in seropositive patients (all *p* < 0.05). **Conclusion:** *Borrelia burgdorferi* IgG seropositivity in patients with ulcerative colitis was associated with lower laboratory markers of systemic inflammation and a more stable hematological profile. Although causality cannot be established, these findings may suggest a potential association between prior Borrelia exposure and a distinct inflammatory phenotype in UC; however, this relationship should be interpreted with caution. Further prospective and mechanistic studies are warranted to clarify the potential immunological interactions between environmental microbial exposure and inflammatory bowel disease activity.

## 1. Introduction

Ulcerative colitis (UC) is a chronic inflammatory bowel disease characterized by relapsing and remitting inflammation confined to the colonic mucosa. Although its exact etiopathogenesis has not been fully elucidated, genetic susceptibility, environmental factors, microbial exposures, and dysregulated immune responses are recognized as key contributors to disease development. In recent years, increasing attention has been directed toward the potential role of infectious agents in modulating host immune responses and influencing the clinical course and disease activity of inflammatory bowel diseases.

*Borrelia burgdorferi* is a zoonotic spirochete transmitted by Ixodes ticks and is the causative agent of Lyme disease. Early infection typically presents with erythema migrans and systemic symptoms, whereas untreated or persistent infection may lead to late complications such as chronic arthritis, neurological involvement, and cardiac conduction abnormalities. Epidemiologically, Lyme disease is most prevalent in North America and Northern Europe. In the United States, more than 30,000 cases are reported annually, making it the most common vector-borne disease [1]. In Europe, more than 65,000 cases are reported each year [2]. In Turkey, particularly in northern regions such as the Black Sea coast, notable seropositivity rates have been documented; although national prevalence data are limited, reported seropositivity rates vary between 2% and 35% across different regions [3].

One of the most remarkable features of *Borrelia burgdorferi* infection is its ability to initiate a strong immune response while simultaneously modulating host immunity in a manner that may promote persistence. Experimental studies have shown delayed B-cell responses and limited development of long-lived plasma cells and memory B cells during infection [4]. In addition, IL-10 has been demonstrated to inhibit *B. burgdorferi*–induced IL-17 production and attenuate IL-17–mediated inflammation [5]. More recent data indicate that infection induces the PD-1/PD-L1 pathway, limiting T-cell infiltration without impairing bacterial clearance [6]. Furthermore, interactions between Borrelia and human dendritic cells have been shown to reshape adaptive immune responses [7]. Suppression of humoral immunity during infection may also vary over time [8]. This multilayered immunomodulation may create a biological environment capable of influencing the course of concurrent inflammatory or autoimmune conditions.

Ulcerative colitis is primarily characterized by cytokine imbalance, altered T-cell polarization, and sustained activation of the mucosal immune response. In this context, it is biologically plausible that a pathogen with chronic immunomodulatory potential, such as *Borrelia burgdorferi*, could influence inflammatory activity in patients with UC. However, to date, no study has systematically evaluated the seroprevalence of *Borrelia burgdorferi* in patients with ulcerative colitis or examined the relationship between seropositivity and disease activity.

The present study aimed to determine the seroprevalence of *Borrelia burgdorferi* IgG antibodies in patients with ulcerative colitis and to assess the association between seropositivity and clinical and laboratory markers of disease activity. To the best of our knowledge, this is the first retrospective observational study investigating the relationship between *Borrelia burgdorferi* exposure and ulcerative colitis.

## 2. Materials and Methods

### 2.1. Study Design and Population

This study was designed as a single-center, retrospective observational study. A total of 100 patients with a confirmed diagnosis of ulcerative colitis (UC) who were followed at the Gastroenterology Department of Giresun University Faculty of Medicine, Türkiye, between October 2020 and October 2024 were included in this study. Ulcerative colitis diagnosis was established based on clinical, endoscopic, and histopathological findings consistent with established inflammatory bowel disease diagnostic criteria. Most participants were residents of the Black Sea region of Türkiye and were of Turkish ethnic origin, representing a relatively homogeneous regional population with potential environmental exposure to tick-borne pathogens. These patients had undergone *Borrelia burgdorferi* IgG serological testing due to musculoskeletal complaints (such as arthralgia or arthritis) and/or neurological symptoms evaluated in physical therapy or neurology clinics, raising suspicion of possible Lyme disease.

Additionally, 145 non-IBD patients who had undergone *Borrelia burgdorferi* serological testing at the same institution during the same period for similar clinical indications were included as a control group. It should be emphasized that this control group does not represent a general healthy population, but rather a clinically selected group of patients who underwent testing for suspected Lyme disease.

### 2.2. Inclusion and Exclusion Criteria

Patients aged 18 years or older with a confirmed diagnosis of ulcerative colitis who had undergone *Borrelia burgdorferi* IgG serological testing due to musculoskeletal or neurological symptoms were eligible for inclusion.

Patients with incomplete clinical or laboratory data, concurrent active infections, a history of malignancy, or other systemic inflammatory or autoimmune diseases were excluded from the study.

### 2.3. Data Collection

Demographic characteristics (age and sex), clinical presentation, laboratory findings, and biochemical parameters were retrospectively obtained from the hospital electronic medical record system. Routine hematological parameters, biochemical tests, inflammatory markers, and thyroid function tests were compared between *Borrelia burgdorferi* IgG seropositive and seronegative groups.

### 2.4. Laboratory Analysis

Serum *Borrelia burgdorferi* IgG antibodies were measured using enzyme immunoassay (EIA) and chemiluminescent immunoassay (CLIA) methods on Triturus and VirClia analyzers (Grifols, Barcelona, Spain). Results were interpreted according to the manufacturer’s instructions as negative (<0.9), borderline (0.9–1.1), and positive (>1.1). Only IgG serology was evaluated in this study; IgM results were not included in the analysis.

### 2.5. Assessment of Disease Activity

Disease activity in patients with ulcerative colitis was assessed based on available clinical findings and laboratory parameters. Due to the retrospective design of the study, validated clinical activity indices such as the Truelove and Witts criteria or the Mayo score could not be systematically calculated for all patients. Therefore, C-reactive protein (CRP) levels and inflammation-related biochemical and hematological parameters were used as indirect indicators of disease activity. Differences in laboratory parameters between *Borrelia burgdorferi* IgG seropositive and seronegative groups were analyzed in relation to potential disease activity.

### 2.6. Statistical Analysis

Statistical analyses were performed using SPSS software (IBM SPSS Statistics for Windows, Version 26.0, IBM Corp., Armonk, NY, USA). The distribution of continuous variables was assessed using the Kolmogorov–Smirnov test. In addition, normality assumptions were verified prior to selecting appropriate parametric or non-parametric tests. Normally distributed variables were expressed as mean ± standard deviation and compared using the independent samples *t*-test. Non-normally distributed variables were presented as median (minimum–maximum) or mean ± standard deviation and compared using the Mann–Whitney U test. Categorical variables were expressed as numbers and percentages and analyzed using the chi-square test. A *p*-value < 0.05 was considered statistically significant.

## 3. Results

### 3.1. Baseline Characteristics of the Study Population

A total of 100 patients with ulcerative colitis and 145 non-IBD controls were included in the study. The mean age of UC patients was 48.5 ± 17 years, while the mean age of the control group was 50.1 ± 16.3 years. In the UC cohort, 59 (59%) patients were male and 41 (41%) were female. In the control group, 51 (35.2%) were male and 94 (64.8%) were female. *Borrelia burgdorferi* IgG seropositivity was detected in 22 (22%) UC patients and 22 (15.2%) control subjects. The baseline characteristics of the study population are summarized in Table 1.

### 3.2. Non-IBD Patients (Control Group)

The non-IBD control group consisted of 145 patients, of whom 94 (64.8%) were female and 51 (35.2%) were male, with a mean age of 50.1 ± 16.3 years. *Borrelia burgdorferi* IgG seropositivity was detected in 22 patients (15.2%).

The mean age of seropositive patients was significantly higher than that of seronegative patients (*p* = 0.037). No statistically significant differences were observed between the groups in terms of sex distribution or other laboratory parameters (all *p* > 0.05).

The comparative demographic and laboratory characteristics of the control group are presented in Table 2.

### 3.3. Ulcerative Colitis Patients

A total of 100 patients with a confirmed diagnosis of ulcerative colitis who were evaluated between October 2020 and October 2024 due to accompanying musculoskeletal (arthralgia/arthritis) and/or neurological symptoms were included in the study. Of these patients, 41 (41%) were female and 59 (59%) were male, with a mean age of 48.5 ± 17 years.

*Borrelia burgdorferi* IgG seropositivity was detected in 22 (22%) patients with ulcerative colitis. No statistically significant difference was observed between seropositive and seronegative patients in terms of sex distribution (12 males/10 females; *p* > 0.05). Similarly, there was no significant difference in mean age between the two groups (50.8 ± 19.7 vs. 47.2 ± 16.2 years; *p* = 0.384).

In terms of overall mean age, the non-IBD control group was numerically older than the ulcerative colitis group (50.1 ± 16.3 vs. 48.5 ± 17 years).

### 3.4. Comparison of Laboratory Findings

When laboratory parameters were compared between *Borrelia burgdorferi* IgG seropositive and seronegative patients within the ulcerative colitis group, uric acid, C-reactive protein (CRP), and alkaline phosphatase levels were found to be significantly lower in the seropositive group (*p* = 0.001, *p* = 0.020, and *p* = 0.023, respectively).

In addition, free T4, mean corpuscular volume (MCV), and mean corpuscular hemoglobin (MCH) values were significantly higher in seropositive patients, whereas RDW-CV values were significantly lower (all *p* < 0.05).

No statistically significant differences were observed between the groups with respect to other hematological, biochemical, or electrolyte parameters (all *p* > 0.05). The complete comparative demographic and laboratory data for the ulcerative colitis cohort are presented in Table 3.

## 4. Discussion

In this single-center retrospective study, we investigated the association between *Borrelia burgdorferi* (BB) IgG seropositivity and associated clinical and laboratory characteristics in patients with ulcerative colitis (UC). The seroprevalence of BB IgG in the UC cohort was 22%, which was numerically higher than the 15.2% observed in the non-IBD control cohort; however, this difference did not reach statistical significance. Because this study evaluated the association between Borrelia IgG seropositivity and disease-related laboratory parameters within the UC population, no age-adjusted multivariate comparison between UC and control groups was performed. Larger population-based studies are required to clarify whether UC patients truly exhibit altered Borrelia exposure rates. Accordingly, the primary aim of this study was to explore the association between Borrelia seropositivity and laboratory-based indicators of inflammatory activity, rather than validated clinical disease activity indices, within a selected UC population.

It should also be considered that the control group consisted of patients who underwent Borrelia testing based on clinical suspicion of Lyme disease and therefore may not represent a general population reference group.

In the UC cohort, BB IgG seropositive patients demonstrated significantly lower CRP, uric acid, and alkaline phosphatase (ALP) levels compared to seronegative patients. CRP is a well-established biomarker of systemic inflammation in ulcerative colitis and correlates with inflammatory activity and mucosal inflammation [9]. Therefore, the lower CRP levels observed in the seropositive group may reflect a distinct inflammatory profile within this subgroup. Similarly, ALP is increasingly recognized not only as a hepatobiliary enzyme but also as a marker associated with intestinal epithelial integrity and mucosal inflammatory activity. Experimental studies have demonstrated anti-inflammatory and barrier-protective roles of intestinal alkaline phosphatase [10]. In this context, the lower ALP levels in seropositive UC patients may indirectly suggest a milder inflammatory state.

From a hematological perspective, BB IgG seropositive UC patients exhibited higher MCV and MCH values and lower RDW-CV levels. Increased RDW has previously been associated with active inflammatory bowel disease [11] and is thought to reflect inflammation-driven ineffective erythropoiesis mediated through IL-6–induced hepcidin upregulation and disturbed iron metabolism [12]. Accordingly, the lower RDW-CV values in the seropositive group may indicate a more stable erythropoietic environment and reduced inflammatory burden. The relatively higher MCV and MCH values in this group are biologically consistent with this interpretation.

Another notable finding was the slightly higher free T4 levels in BB IgG seropositive UC patients, while TSH levels did not differ significantly. Chronic inflammatory states are known to influence thyroid hormone metabolism, sometimes resulting in non-thyroidal illness–like patterns. The lower CRP levels in the seropositive group may explain the relatively preserved thyroid hormone profile observed in these patients. However, this association should be interpreted cautiously and requires confirmation in prospective studies.

Mechanistically, *Borrelia burgdorferi* has been shown to modulate host immunity through multiple pathways. Experimental studies indicate that BB infection can alter B-cell responses, limit long-lived plasma cell development, and shape adaptive immune responses [4]. IL-10–mediated suppression of IL-17 production has also been described in the context of Borrelia infection [5]. Furthermore, induction of the PD-1/PD-L1 axis during infection may regulate T-cell infiltration without impairing bacterial clearance [6]. Interactions with dendritic cells and dynamic suppression of humoral immunity over time have also been demonstrated [7,8]. Collectively, these findings suggest that Borrelia exposure may induce a complex and regulated immune environment rather than a purely proinflammatory response.

In this context, BB IgG seropositivity in UC patients should not be interpreted as evidence of active infection or a direct anti-inflammatory effect. Rather, it may be associated with a distinct immune-regulatory profile that coincides with lower laboratory markers of inflammation. Whether this reflects immune adaptation, prior antigenic exposure, or host-specific immunological traits remains unclear. The present findings should therefore be considered hypothesis-generating rather than causal. These findings should be interpreted with caution, as the retrospective design and lack of temporal data preclude any causal inference regarding the relationship between Borrelia exposure and ulcerative colitis.

Although IgG-dominant immune responses—such as those induced by natural infections or protein-based immunogens—can sometimes be associated with more regulated immune states, no clinical evidence currently supports a therapeutic implication of Borrelia-related immune responses in inflammatory bowel disease. Any extrapolation toward immunomodulatory strategies remains speculative and requires rigorous mechanistic and prospective validation.

## 5. Limitations

This study has several limitations. First, its retrospective and single-center design limits generalizability. Additionally, the study population represents a selected subgroup of patients who underwent Borrelia serological testing due to specific clinical indications (musculoskeletal or neurological symptoms), which may introduce selection bias and limit generalizability to the broader UC population. It should also be noted that Borrelia serology is not routinely performed in patients with ulcerative colitis; therefore, only patients who presented to neurology or physical therapy clinics with relevant symptoms and underwent serological testing due to clinical suspicion of Lyme disease were included in the analysis. Furthermore, differences in age and sex distribution between groups may act as potential confounding factors. Second, BB IgG seropositivity reflects prior exposure rather than active infection, and IgM or confirmatory immunoblot analyses were not systematically evaluated. Additionally, confirmatory testing (e.g., Western blot or two-tier testing) was not systematically available in the dataset due to the retrospective study design, which may have introduced false-positive serological results. Third, validated clinical activity indices such as the Mayo score were not uniformly available; therefore, laboratory parameters were used as surrogate markers of disease activity. Fourth, no multivariate age-adjusted comparison between UC and non-IBD cohorts was performed, as the primary aim focused on within-UC associations. Finally, the relatively small sample size, particularly in the seropositive subgroup, limits statistical power and precludes definitive conclusions.

## 6. Conclusions

*Borrelia burgdorferi* IgG seropositivity in patients with ulcerative colitis was associated with lower laboratory markers of systemic inflammation and a more stable hematological profile. While these findings do not establish causality, they may suggest a potential association between prior Borrelia exposure and a distinct inflammatory phenotype in UC; however, this relationship should be interpreted with caution. These observations are hypothesis-generating and warrant further prospective and mechanistic investigations to clarify the immunological interplay between environmental microbial exposure and inflammatory bowel disease activity.

## Figures and Tables

**Table 1:** Baseline demographic characteristics of the study population.

Parameter	UC Patients (*n* = 100)	Controls (*n* = 145)
Age (mean ± SD)	48.5 ± 17	50.1 ± 16.3
Male, *n* (%)	59 (59%)	51 (35.2%)
Female, *n* (%)	41 (41%)	94 (64.8%)
Borrelia IgG positive, *n* (%)	22 (22%)	22 (15.2%)

**Table 2:** Demographic and laboratory characteristics of non-IBD patients stratified by *Borrelia burgdorferi* IgG serostatus.

Parameter	Negative (*n* = 123) Mean ± SD	Positive (*n* = 22) Mean ± SD	*p* Value
Age (years)	48.2 ± 15.4	60.8 ± 17.4	*p* = 0.037 *
WBC (×10^3^/µL)	7.8 ± 2.6	6.9 ± 1.4	*p* = 0.277
Hemoglobin (g/dL)	13.1 ± 1.7	13.3 ± 1.9	*p* = 0.657
Hematocrit (%)	39.2 ± 4.6	39.7 ± 4.8	*p* = 0.219
MCV (fL)	86.5 ± 6.1	86.0 ± 6.3	*p* = 0.933
MCHC (g/dL)	32.9 ± 1.0	32.9 ± 1.1	*p* = 0.526
Neutrophils (×10^3^/µL)	5.0 ± 2.5	4.2 ± 1.1	*p* = 0.487
Lymphocytes (×10^3^/µL)	2.1 ± 0.8	2.1 ± 0.7	*p* = 0.629
Platelets (×10^3^/µL)	264 ± 77	256 ± 68	*p* = 0.817
Glucose (mg/dL)	106.1 ± 31.7	110.8 ± 24.1	*p* = 0.182
Urea (mg/dL)	30.3 ± 16.9	29.6 ± 10.8	*p* = 0.684
Creatinine (mg/dL)	0.7 ± 0.3	0.7 ± 0.2	*p* = 0.851
AST (U/L)	27.3 ± 55.2	20.0 ± 7.8	*p* = 0.512
ALT (U/L)	24.8 ± 38.4	18.6 ± 11.5	*p* = 0.510
Albumin (g/dL)	4.3 ± 0.5	4.4 ± 0.3	*p* = 0.763
ALP (U/L)	85.2 ± 42.2	71.0 ± 19.8	*p* = 0.510
GGT (U/L)	27.3 ± 27.7	22.4 ± 10.8	*p* = 0.965
Sodium (mmol/L)	139.5 ± 2.6	140.0 ± 2.0	*p* = 0.941
Potassium (mmol/L)	4.4 ± 0.4	4.4 ± 0.3	*p* = 0.922
Chloride (mmol/L)	102.3 ± 2.7	102.4 ± 2.8	*p* = 0.494
Calcium (mg/dL)	9.4 ± 0.6	9.5 ± 0.5	*p* = 0.877
TSH (µIU/mL)	2.0 ± 1.2	2.0 ± 1.1	*p* = 0.797
Free T4 (ng/dL)	1.2 ± 0.4	1.2 ± 0.2	*p* = 0.459
Male, *n* (%)	41 (33.3%)	6 (27.3%)	*p* = 0.576 †
Female, *n* (%)	82 (66.7%)	16 (72.7%)	—

* *p* < 0.05 was considered statistically significant; † Chi-square test; — Not applicable.

**Table 3:** Comparison of demographic and laboratory parameters according to *Borrelia burgdorferi* IgG serostatus in patients with ulcerative colitis.

Parameter	Negative (Mean ± SD)	Positive (Mean ± SD)	Test	*p* Value
Age (years)	47.18 ± 16.24	50.86 ± 19.68	Mann–Whitney U	*p* = 0.384
Glucose (mg/dL)	102.0 ± 32.2	105.3 ± 42.5	Mann–Whitney U	*p* = 0.772
Urea (mg/dL)	28.40 ± 9.35	28.84 ± 9.43	Independent *t*-test	*p* = 0.850
eGFR (mL/min/1.73 m^2^)	77.07 ± 28.85	96.85 ± 30.33	Mann–Whitney U	*p* = 0.438
Creatinine (mg/dL)	0.84 ± 0.18	0.85 ± 0.14	Mann–Whitney U	*p* = 0.780
ALT (U/L)	21.93 ± 38.74	28.84 ± 58.13	Mann–Whitney U	*p* = 0.643
AST (U/L)	19.46 ± 6.82	27.0 ± 36.95	Mann–Whitney U	*p* = 0.983
Total Bilirubin (mg/dL)	0.52 ± 0.47	0.58 ± 0.36	Mann–Whitney U	*p* = 0.191
Direct Bilirubin (mg/dL)	0.19 ± 0.11	0.22 ± 0.11	Mann–Whitney U	*p* = 0.098
Indirect Bilirubin (mg/dL)	0.33 ± 0.37	0.32 ± 0.17	Mann–Whitney U	*p* = 0.323
Total Protein (g/L)	71.58 ± 5.44	72.41 ± 5.65	Mann–Whitney U	*p* = 0.744
Albumin (g/L)	42.67 ± 7.54	45.17 ± 3.33	Mann–Whitney U	*p* = 0.210
Uric Acid (mg/dL)	5.60 ± 1.35	4.38 ± 1.27	Independent *t*-test	*p* = 0.001 *
Alkaline Phosphatase (U/L)	100.8 ± 73.9	89.2 ± 68.2	Mann–Whitney U	*p* = 0.023 *
GGT (U/L)	27.62 ± 41.33	19.30 ± 15.55	Mann–Whitney U	*p* = 0.192
LDH (U/L)	184.7 ± 42.3	193.7 ± 44.29	Mann–Whitney U	*p* = 0.292
Sodium (mmol/L)	139.6 ± 2.17	139.6 ± 2.83	Mann–Whitney U	*p* = 0.404
Potassium (mmol/L)	4.37 ± 0.42	4.45 ± 0.47	Mann–Whitney U	*p* = 0.928
Calcium (mg/dL)	9.49 ± 0.44	9.61 ± 0.46	Mann–Whitney U	*p* = 0.449
Magnesium (mg/dL)	2.03 ± 0.16	2.05 ± 0.11	Mann–Whitney U	*p* = 0.633
Phosphorus (mg/dL)	3.40 ± 0.63	3.29 ± 0.51	Mann–Whitney U	*p* = 0.557
Amylase (U/L)	70.68 ± 26.37	79.40 ± 33.47	Mann–Whitney U	*p* = 0.405
CRP (mg/L)	10.50 ± 16.76	4.83 ± 7.96	Mann–Whitney U	*p* = 0.020 *
Free T4 (ng/dL)	1.13 ± 0.23	1.24 ± 0.16	Mann–Whitney U	*p* = 0.049 *
TSH (µIU/mL)	2.27 ± 1.85	2.03 ± 1.61	Mann–Whitney U	*p* = 0.453
Ferritin (ng/mL)	93.39 ± 127.12	69.87 ± 55.02	Mann–Whitney U	*p* = 0.813
Folate (ng/mL)	6.54 ± 3.20	6.75 ± 3.08	Mann–Whitney U	*p* = 0.894
Vitamin B12 (pg/mL)	393.6 ± 192.0	411.0 ± 182.4	Mann–Whitney U	*p* = 0.780
WBC (×10^3^/µL)	7.73 ± 2.42	6.88 ± 1.31	Mann–Whitney U	*p* = 0.267
Neutrophils (×10^3^/µL)	4.68 ± 2.06	4.06 ± 1.15	Mann–Whitney U	*p* = 0.402
Neutrophils (%)	58.6 ± 10.5	58.5 ± 8.85	Independent *t*-test	*p* = 0.980
Lymphocytes (×10^3^/µL)	2.26 ± 0.87	2.02 ± 0.62	Mann–Whitney U	*p* = 0.299
Lymphocytes (%)	30.28 ± 9.91	29.59 ± 8.13	Independent *t*-test	*p* = 0.765
Monocytes (×10^3^/µL)	0.62 ± 0.18	0.55 ± 0.13	Independent *t*-test	*p* = 0.106
Monocytes (%)	8.25 ± 1.98	8.21 ± 2.11	Independent *t*-test	*p* = 0.942
Eosinophils (×10^3^/µL)	0.17 ± 0.12	0.19 ± 0.14	Mann–Whitney U	*p* = 0.557
Eosinophils (%)	2.36 ± 1.76	3.39 ± 2.65	Mann–Whitney U	*p* = 0.110
RBC (×10^6^/µL)	4.75 ± 0.49	4.64 ± 0.41	Independent *t*-test	*p* = 0.350
Hemoglobin (g/dL)	13.12 ± 1.98	13.52 ± 1.55	Independent *t*-test	*p* = 0.388
Hematocrit (%)	40.44 ± 4.83	41.04 ± 3.85	Independent *t*-test	*p* = 0.598
MCV (fL)	85.24 ± 6.84	88.42 ± 4.06	Mann–Whitney U	*p* = 0.032 *
MCH (pg)	27.63 ± 3.07	29.17 ± 2.19	Mann–Whitney U	*p* = 0.025 *
MCHC (g/dL)	32.34 ± 1.49	32.92 ± 1.62	Independent *t*-test	*p* = 0.119
RDW-SD (fL)	44.60 ± 5.69	43.34 ± 4.14	Mann–Whitney U	*p* = 0.446
RDW-CV (%)	14.54 ± 2.39	13.45 ± 1.57	Mann–Whitney U	*p* = 0.030 *
Platelets (×10^3^/µL)	298.7 ± 91.8	283.0 ± 85.2	Mann–Whitney U	*p* = 0.325
PCT (%)	0.29 ± 0.08	0.27 ± 0.07	Mann–Whitney U	*p* = 0.446
MPV (fL)	9.78 ± 0.80	9.90 ± 0.79	Independent *t*-test	*p* = 0.562
PDW (fL)	10.91 ± 1.81	11.47 ± 1.95	Mann–Whitney U	*p* = 0.091
P-LCR (%)	23.55 ± 6.32	24.45 ± 6.29	Independent *t*-test	*p* = 0.566
Lipase (U/L)	39.11 ± 27.07	37.57 ± 17.55	Mann–Whitney U	*p* = 0.832
Free T3 (pg/mL)	3.19 ± 0.46	3.36 ± 0.47	Independent *t*-test	*p* = 0.179
Chloride (mmol/L)	103.4 ± 3.10	103.6 ± 2.44	Mann–Whitney U	*p* = 0.795

* *p* < 0.05 was considered statistically significant.

## Data Availability

The datasets generated and/or analyzed during the current study are not publicly available due to institutional regulations and patient confidentiality, but are available from the corresponding author on reasonable request.

## Abbreviations

ALP, alkaline phosphatase; ALT, alanine aminotransferase; AST, aspartate aminotransferase; BB, *Borrelia burgdorferi*; CRP, C-reactive protein; eGFR, estimated glomerular filtration rate; GGT, gamma-glutamyl transferase; Hct, hematocrit; Hgb, hemoglobin; IBD, inflammatory bowel disease; IgG, immunoglobulin G; MCV, mean corpuscular volume; MCH, mean corpuscular hemoglobin; MCHC, mean corpuscular hemoglobin concentration; MPV, mean platelet volume; NK, natural killer; PDW, platelet distribution width; PLT, platelet count; RDW, red cell distribution width; RDW-CV, red cell distribution width–coefficient of variation; sT3, free triiodothyronine; sT4, free thyroxine; TSH, thyroid-stimulating hormone; UC, ulcerative colitis; WBC, white blood cell count.
